# Clinical features of hereditary transthyretin amyloidosis-polyneuropathy with transthyretin Ala97Ser(p.Ala117Ser) mutation in South Mainland China

**DOI:** 10.1186/s13023-025-03733-0

**Published:** 2025-04-28

**Authors:** Yeli Zhu, Jingxian Fan, Xiying Zhu, Wei Li, Zhaoyong Zhang, Hui Zheng, Zhihua Zhou, Lingchao Meng, Ruxu Zhang, Haishan Jiang

**Affiliations:** 1 Department of Neurology, Southern Medical University Nanfang Hospital, Southern Medical University, Guangzhou, China; 2 Department of Neurology, Central South University Third Xiangya Hospital, Central South University, Changsha, China; 3 Department of Neurology, Peking University First Hospitall, Peking University, Beijing, China; 4 Department of Neurology, The First affiliated Hospital of Guangdong Pharmaceutical University, Guangzhou, China

**Keywords:** Hereditary transthyretin amyloidosis (ATTRv), *TTR* gene mutation, Clinical feature, Scoring model

## Abstract

**Objective:**

Our study aimed to report the clinical features and epidemiological characteristics of hereditary transthyretin amyloidosis-polyneuropathy(ATTRv-PN) with *TTR* Ala97Ser(p.Ala117Ser) mutation from South Mainland China.

**Methods:**

We identified 21 patients from 20 families diagnosed with Ala97Ser ATTRv-PN based on strict clinical and electrophysiological criteria from three centers. Clinical and laboratory data were retrospectively retrieved for analysis.

**Results:**

A gender imbalance was noted with a male-to-female ratio of 18:3. All patients showed late onset, with the age of onset at 56.5 ± 7.2 years. The predominant initial symptom, reported by 15 patients (71.4%), was numbness. Paraesthesia was present in all patients. Eighteen patients (85.7%) had autonomic dysfunction. Cardiac, renal, and ocular dysfunctions were noted in 17 (80.9%), 4(19.0%), and 4(19.0%) patients, respectively. Nerve conduction studies have shown axonal-type sensorimotor polyneuropathy. The decline in sensory nerve action potentials was more noticeable than in compound muscle action potentials. The nerve damage present in the lower limbs was more severe than that in the upper limbs. Nerve biopsy revealed positive Congo red staining in 11/15 patients (73.3%).

**Conclusion:**

ATTRv-PN appears relatively rare in South Mainland China, with our study providing the largest cohort of Ala97Ser mutation cases to date. We found a significant founder effect by combining the clinical and demographic characteristics. That helps us understand the gene’s transmission pathway and lays the foundation for carrier screening and tertiary prevention and control. We also propose a new scoring model and demonstrate that this model allows the profiling of different genotypes of ATTRv-PN, facilitating early clinical detection and diagnosis.

**Supplementary Information:**

The online version contains supplementary material available at 10.1186/s13023-025-03733-0.

## Introduction

Hereditary transthyretin amyloidosis-polyneuropathy (ATTRv-PN) is an autosomal dominant genetic disorder caused by point mutations of the *TTR* gene. It is characterized by amyloid deposition in many systems including the peripheral and autonomic nervous system [1]. It is a systemic disease that involves various organs (such as the heart, eyes, and kidney) and usually presents as progressive peripheral neuropathy with adult-onset. In 1952, Andrade first described ATTRv-PN in northern Portugal [2]. It was subsequently reported in Japan (1968) [3] and Sweden (1976) [4]. ATTRv-PN has been reported in 29 countries, including Korea [5], the United States of America [6], China [7], Thailand [8], and many European countries [9].

The *TTR* gene is located on chromosome 18 and comprises four exons [10]. More than 130 mutations have been identified associated with this gene [1]. The Val30Met (p.Val50Met) variant of *TTR* is most commonly identified among patients living in small clusters and scattered families worldwide and was first described as the cause of ATTRv-PN in 1984 [11]. Moreover, certain specific mutations are associated with small clusters of families in particular areas. For example, the *TTR* Ala97Ser (p.Ala117Ser) mutation is common among Chinese kindreds from the Taiwan area [12–15]. However, this mutation seems to be mainly distributed in South Mainland China. Subsequently, the first ATTRv-PN family with a proven missense mutation c.349G > T Ala97Ser in Southern Mainland China was reported in 2018 [16]. Through investigating several ATTRv-PN centers in China, we found a founder effect in patients with Ala97Ser mutation in South China, and it may be related to patients in the Taiwan area. The study aimed to document the distribution features of ATTRv-PN with Ala97Ser mutation and summarize its characteristic clinical manifestations. ATTRv-PN appears relatively rare in China, while our research provides the largest cohort of Ala97Ser mutation cases to date.

## Subjects and methods

### Patients

We identified 21 patients from 20 distinct families diagnosed with Ala97Ser ATTRv-PN, based on strict clinical and electrophysiological criteria from the Department of Neurology of three centers: Southern Medical University Nanfang Hospital, Peking University First Hospital, Central South University Third Xiangya Hospital, September 2013 to September 2024. All patients signed an informed consent form prior to inclusion in the study. All patients conformed to the latest diagnostic criteria for ATTRv-PN, particularly the typical pathological features of the nerve biopsy, mutations in the *TTR* Ala97Ser gene, and the exclusion of other diseases. Furthermore, the clinical and laboratory data were retrieved for analysis. Procedures of the tests (nerve conduction study(NCS), ultrasonic cardiogram (UCG), cerebrospinal fluid (CSF) examination, routine blood and urine examinations, genetic analysis, nerve biopsies, etc.) followed established protocols.

### TTR gene analysis

Peripheral venous blood samples were obtained for DNA analysis from the patients. Genomic DNA was isolated from the blood samples following a standard protocol. Briefly, the four exons of the entire human *TTR* gene (NCBI Reference Sequence: NG_009490.1, NM_000371.3) were amplified by polymerase chain reaction (PCR). The PCR products were purified and sequenced directly by Sanger sequencing.

### Electrophysiologic assessment

Neuroelectrophysiological assessment was done in all patients following standard procedures with surface stimulating and recording electrodes, including nerve conduction studies (orthodromic recording) of motor and sensory nerves at the lower and upper limbs in combination with the test of F wave. Motor conduction was investigated in the median, ulnar, tibial, and common peroneal nerve. Sensory conduction was investigated in the median, ulnar, superficial fibular, and sural nerves.

### Nerve biopsy and pathological assessment

A sural nerve (or sensory branch of the superficial peroneal nerve) biopsy was performed under local skin and tissue anesthesia, excluding the nerve. Nerve specimens were processed for routine stains (hematoxylin-eosin for overview and nerve morphology; Staining with TTR antibody and Congo red for amyloid) on frozen and semi-thin sections (azure-methylene blue). Serial consecutive sections were assessed. Electron microscopy samples were fixed in a 2.5% glutaraldehyde buffer for 2 h, then with osmium acid, dehydrated in acetone, and embedded with epoxy resin. The sections were observed and photographed under an electron microscope.

### Multisystem evaluation

Subsequently, we facilitated the diagnostic process through a comprehensive assessment of pathological changes in multiple organ systems, including the peripheral nervous system, autonomic nervous system, cardiovascular system, renal system, and ocular system. Peripheral neuropathy is assessed through neurological examination, electromyography (EMG), and nerve biopsy to quantify axonal or demyelinating damage. Autonomic dysfunction is evaluated via symptom profiling (e.g., orthostatic intolerance, gastrointestinal dysmotility) combined with orthostatic blood pressure testing to identify hemodynamic instability. Cardiac involvement requires multimodal imaging and functional analyses, including electrocardiography (ECG) for arrhythmia detection, echocardiography (Echo) to assess chamber dimensions and ejection fraction, and myocardial Tc-99 m Pyrophosphate (PYP) Scintigraphy to delineate amyloid deposition or perfusion defects. Renal function is systematically monitored through serial measurements of serum creatinine (Scr) and urinary protein quantification (e.g., 24-hour proteinuria or urine protein-to-creatinine ratio) to stage chronic kidney disease. Ocular pathology is investigated by correlating subjective visual complaints (e.g., scotomas, blurred vision) with objective findings on fundoscopy, such as vitreous opacities or retinal vascular abnormalities, to confirm visual pathway compromise.

### The scoring system

Subsequently, we proceed to score the patients based on the results of the aforementioned multisystem evaluation. For each symptom or abnormality identified in each system as detailed in Supplementary Table 1, one point is assigned.

### Statistical analyses

We used IBM SPSS Version 25 and Microsoft Office Excel. Descriptive statistics are mainly used in this study. Numeric variables are here described as means ± SD, numbers (n) with percentages (%) or median values, etc. Results were considered significant at *p*<0.05.

## Results

### Clinical features

**Fig. 1:**
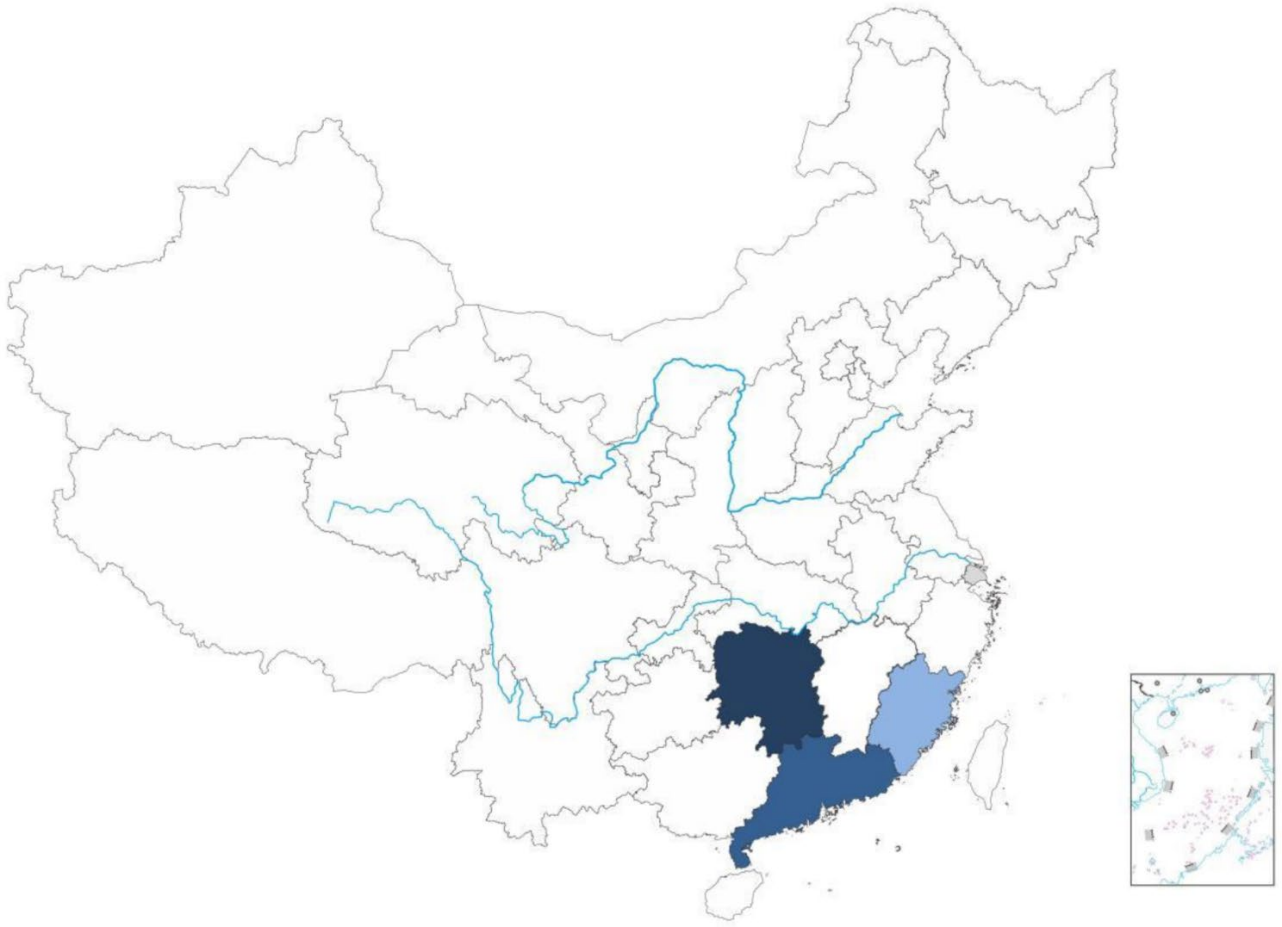
Geographical distribution of the probands with Ala97Ser (p.Ala117Ser) ATTRv-PN. The dark blue region: 10 patients from Hunan province, China. The middle dark blue region: 7 patients fromGuangdong province, China. The light blue region: 3 patients from Fujian province, China. The gray region: 1 patient from Shanghai, China

The basic information of the probands (18 males and 3 females) in the 20 families with Ala97Ser ATTRv-PN is summarised in Table 1. All probands were from South Mainland China (see Fig. 1). The age of onset was 56.5 ± 7.2 years (range 40–66). The period from the onset to the final diagnosis was 4.9 ± 3.9 years (range 0–13), and neurological assessments were performed at the time of diagnosis. Initial symptoms were numbness of the lower or upper extremities (15 patients), weakness of the lower limbs (2 patients), diarrhea and constipation (1 patient), erectile dysfunction (1 patient), Decreased exercise tolerance (1 patient), and chest tightness (1 patient). No cranial nerve defects, such as dysphagia, dysarthria, or hypertrophy of the tongue, were observed in any of the patients.

**Table 1:** The clinical features of the probands with Ala97Ser (p.Ala117Ser) ATTRv-PN

Patient No.	Gender	Nationality	Birthplace	Age (years)		Interval (years)	Initial complaints	Muscle power (MRC), upper/lower limbs		Tissue proof
				Diagnosis	Onset			Distal	Proximal	
1	M	Han	Changsha, Hunan	66	59	7	Numbness of upper extremities	3- / 2	4- / 4	None
2	M	Han	Zhuzhou, Huan	61	58	3	Numbness of limbs	5 / 4+	4+/ 3	None
3	M	Han	Loudi, Hunan	50	47	3	Diarrhea and constipation	5 / 5	5 / 5	Sural N
4	M	Han	Chenzhou, Hunan	66	64	2	Chest tightness	5 / 5	5 / 5	None
5	M	Han	Shanghai	62	58	4	Numbness of upper extremities	1 / 2	3+/3+	Sural N
6	M	Han	Chaozhou, Guangdong	62	56	6	Weakness of lower extremities	1 / 2	4 / 4	Sural N
7	M	Han	Hengyang, Hunan	61	54	7	Erectile dysfunction	4- /4-	4 / 5	Sural N
8	M	Han	Hengyang, Hunan	67	64	3	Numbness of lower extremities	NK/0	4 / 4	Sural N
9	M	Han	Shenzhen, Guangdong	60	52	12	Numbness of upper extremities	5 / 5	5 / 5	Sural N
10	M	Han	Hengyang, Hunan	65	52	13	Numbness of upper extremities	4 / 3	4- / 4-	Sural N
11	M	Han	Shenzhen, Guangdong	59	57	2	Numbness of lower extremities	2 / 0	4- / 4-	Peroneal N
12	F	Han	Jieyang, Guangdong	68	65	3	Numbness of upper extremities	2 / 2	4- / 4-	Peroneal N
13	F	Han	Loudi, Hunan	67	66	1	Numbness of lower extremities	5 / 5	5 / 5	Peroneal N
14	M	Han	Jiangmen, Guangdong	59	57	2	Numbness of lower extremities	4+/4	5/5	Peroneal N
15	M	Han	Chenzhou, Hunan	41	41	0	Numbness of limbs	5/5	5/5	None
16	M	Han	Nanping, Fujian	67	57	10	Numbness of upper extremities	3/2	4/4	Peroneal N
17	M	Han	Nanping, Fujian	40	40	0	Decreased exercise tolerance	5/5	5/5	None
18	F	Han	Fuzhou, Fujian	72	66	6	Weakness of lower extremities	4/3	4/3	None
19	M	Han	Heyuan, Guangdong	65	55	10	Numbness of limbs	5/4	5/4	Peroneal N
20	M	Han	Chaozhou, Guangdong	68	60	8	Numbness of limbs	5-/4+	5-/4+	Sural N
21	M	Han	Xiangtan, Hunan	60	59	1	Numbness of lower extremities	5-/4	5/5	Peroneal N
Average				61.2 ± 8.3	56.5 ± 7.2	4.9 ± 3.9				

M: male. F: female. N: nerve. NK: unknown.None: without biopsy. Sural N: sural nerve. SP N: superficial peroneal nerve

C-: Congo red staining negative. C+: Congo red staining positive

The clinical manifestations are shown in supplementary Table 1. Fifteen patients suffered from muscular weakness and amyotrophy in the upper and lower limbs to varying degrees in a distally accentuated manner. Paraesthesia was noted in all patients. Almost half of the patients experienced pain in the upper or lower limbs, although sensory dissociation was not conspicuous. Decreased tendon reflexes and carpal tunnel syndrome were prominent in most patients. Eighteen patients complained of autonomic dysfunction during the duration of the disease. However, specific symptoms varied from patient to patient. Constipation was observed in 11 patients. Six of the 11 patients complained about alternating occurrences of diarrhea and constipation. Seventeen patients experienced weight loss. Orthostatic hypotension was seen in seven patients. Erectile dysfunction was observed in 9 /18 male patients. Only four patients had hyperhidrosis. Two patients complained of disturbances in urination (urine retention).

**Fig. 2:**
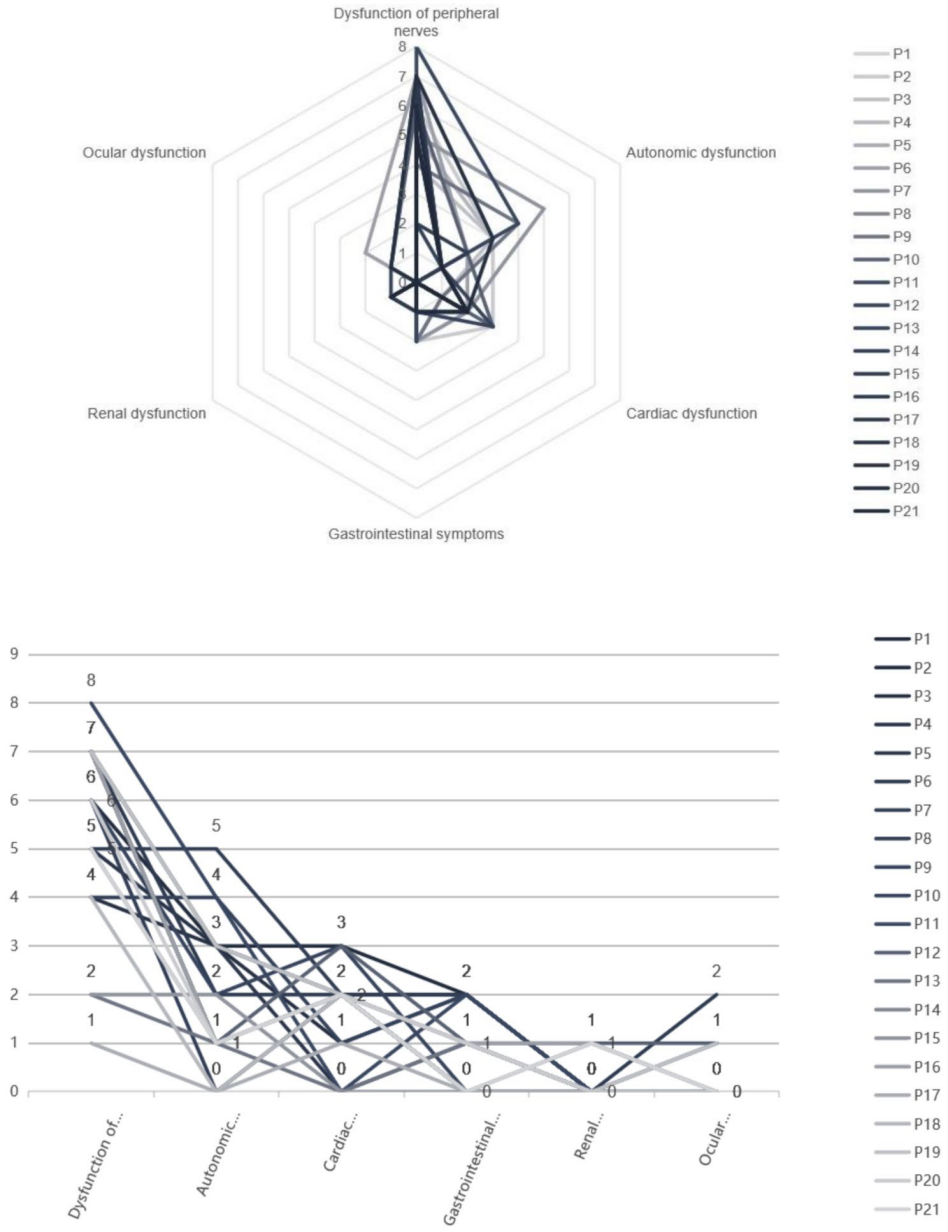
Various systems dysfunction scores of the probands with Ala97Ser (p.Ala117Ser) ATTRv-PN. P1-21: No. 1–21 patient. Radar Map (**a**) and Line Chart (**b**) show the various system dysfunction scores of all patients based on supplementary Table 1. One point for each of the following symptoms: Paresthesia, Sensory dissociation, allodynia, weakness, amyotrophy, decreased reflexes, carpal tunnel syndrome, diarrhea, constipation, weight reduction, orthostatic hypotension, hyperhidrosis, erectile dysfunction, urine retention, arrhythmia, cardiac hypertrophy, symptomatic heart failure, Gastrointestinal symptoms, renal dysfunction, vision loss, vitreous opacity, cataract, glaucoma

Additionally, the involvement of other organs was seen in seventeen patients by means of the multisystemic assessment described in the methods section. Cardiac dysfunction was the most common (17 patients), specifically arrhythmia, cardiac hypertrophy, and symptomatic heart failure. Only 4 patients had renal dysfunction (manifested by abnormal 24-hour urine protein quantification). Four patients experienced ocular dysfunction: decreased vision (4 patients), vitreous opacity (1 patient), and cataract (1 patient). However, none of the patients had glaucoma. Other symptoms included edema (8 patients), dry cough (8 patients), and hemorrhagic rash (2 patients). The various system dysfunction scores of the patients are shown in Fig. 2.

### Neurophysiological manifestations

NCS showed axonal-type sensorimotor polyneuropathy (detailed in Supplementary Table 2). Most patients had long distal latency of the upper or lower extremities, but it was much smaller than in demyelinating neuropathies. All patients had reductions in the amplitudes of compound muscle action potentials (CMAPs) and sensory nerve action potentials (SNAPs) from mild to severe. It is worth noting that the decline in SNAP was more noticeable than that in CMAP. The nerve damage of the lower limbs was more severe than that of the upper limbs. The SNAP of most of the lower extremities was not observed. Additionally, a decrease in the F wave was observed in nearly all tested nerves.

### Histopathological findings

**Fig. 3:**
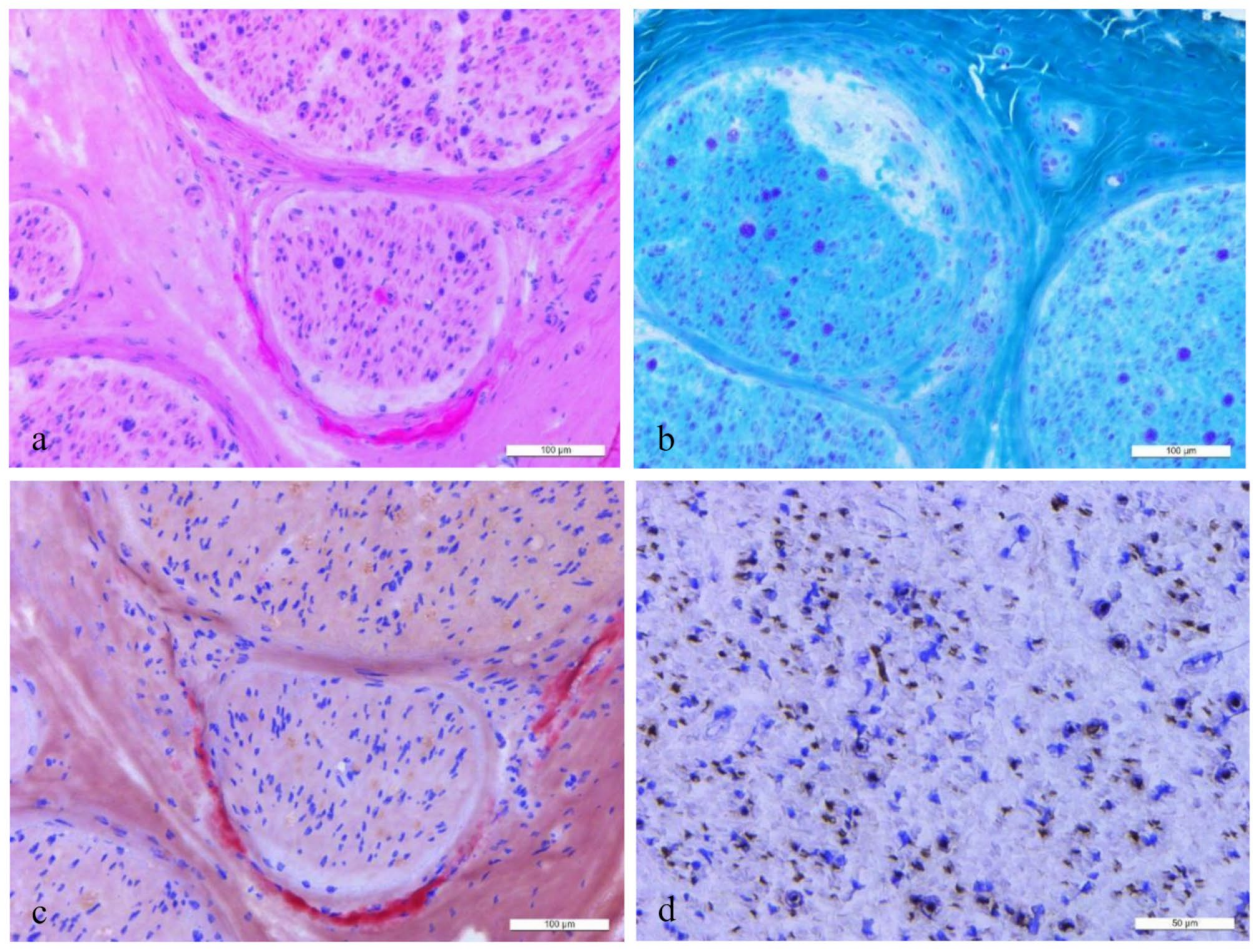
Histopathologic findings of No.10 Patient. HE (**a**) and Congo red staining (**c**) observed multiple red-stained amyloid deposits. MGT staining found axonal degeneration of green substances in some large myelinated fibers (**b**). Moderately to severely decreased unmyelinated nerve fibers in each nerve bundle were shown by NF staining (**d**)

Fifteen patients underwent a sural or peroneal nerve biopsy. The main pathological changes in peripheral nerves were moderate to severely decreased myelinated and unmyelinated nerve fibers, accompanied by degenerative or regenerative changes in the axons and myelin sheath of myelinated nerve fibers, which is following the pathological characteristics of chronic active mixed peripheral neuropathy. Nerve biopsy revealed positive Congo red staining in 11/15 patients (73.3%). Congo red staining showed multiple red-stained amyloid deposits and bright apple-green coloration under a polarising microscope. MGT staining found axonal degeneration of green substances in some large myelinated fibers. The histopathologic findings of patient no. 10 are shown in Fig. 3.

### Tc-99 m pyrophosphate (PYP) scintigraphy

**Fig. 4:**
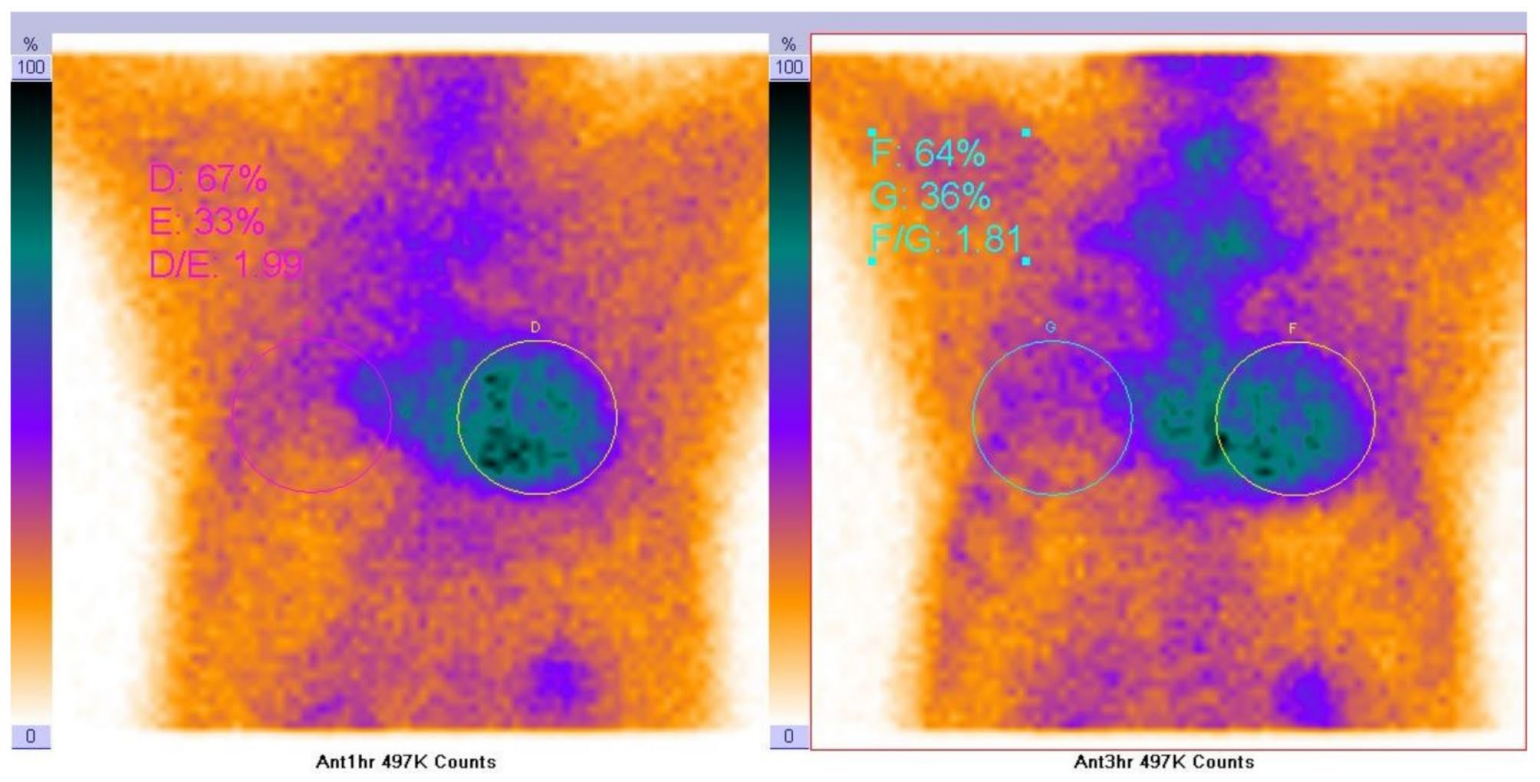
Tc-99 m Pyrophosphate (PYP) Scintigraphy of NO.21 Patient. The result showed tracer retention in the anterior wall (counts were 497 K both at 1 h and 3 h post-injection), and the local uptake ratio was elevated (D/E = 1.99, F/G = 1.81), which is consistent with the imaging features of ATTR amyloid cardiomyopathy

Four patients underwent Tc-99 m Pyrophosphate (PYP) Scintigraphy. Three of them demonstrated positive imaging findings indicative of transthyretin cardiac amyloidosis (ATTR-CA), meeting the 2020 AHA diagnostic criteria for cardiac amyloidosis with Grade 2–3 radiotracer uptake on visual grading. One patient did not fulfill the criteria for ATTR-CA positivity (Grade 0 per 2020 AHA classification). Representative imaging of Patient 21 is illustrated in Fig. 4.

### Misdiagnosis and treatment

Among the 21 patients, 17 patients had previously been misdiagnosed with other diseases. Seven were misdiagnosed with chronic inflammatory demyelinating polyneuropathy (CIDP), four were misdiagnosed with peripheral neuropathies due to other causes, three were misdiagnosed with cervical or lumbar disc herniation, one was misdiagnosed with spinal canal stenosis, and the prior misdiagnosis status of two patients remained unclear. Only four patients had not been misdiagnosed.

Regarding the treatment of patients, almost all patients initially received tafamidis therapy. However, due to cost and other factors, only Twelve patients continued with tafamidis treatment. Additionally, four patients attempted diflunisal therapy but discontinued it due to significant gastrointestinal adverse reactions.

## Discussion

ATTRv-PN appears relatively rare in South Mainland China, while our study provides the largest cohort of Ala97Ser mutation cases. In the 1990s, a family with ATTRv-PN was first diagnosed at the Peking Union Medical College Hospital [17]. It is estimated that there are approximately 1997 cases in China [18]. Over 40 case reports have been published recently, including reports of multiple ATTRv-PN families. Studies describe a mutation in the *TTR* gene, different from that observed in Europe [7, 19–21]. Shortly before, a unicentric retrospective study reported that *TTR* Val30Met remains mainland China’s most common mutation type [22]. However, *TTR* Ala97Ser mutations are relatively rare. Based on previous studies, the *TTR* Ala97Ser mutation was one of the most common variants in the Chinese population, especially in Chinese kindreds from the Taiwan area. This mutation has never been reported in Caucasian populations. Detailed haplotype analyses demonstrated a shared haplotype in most patients with the Ala97Ser mutation in the Taiwan area, suggesting a founder effect [23]. This may be related to the late onset of ATTRv-PN and the early onset of symptoms, which are mild and do not interfere with fertility. Except for the first report of the ATTRv-PN family with a proven *TTR* Ala97Ser mutation from mainland China [16], our study reported another 20 pedigrees with the same mutation. Furthermore, all cases were from south China, especially Hunan and Guangdong provinces. Despite the lack of genetic verification, we speculate that the *TTR* Ala97Ser mutation originated in South Mainland China.

Our study demonstrated the characteristics of ATTRv-PN with the *TTR* Ala97Ser mutation in mainland China. Similar to previous reports (detailed in Table 2), significantly more men than women were diagnosed with this mutation. However, it is unclear whether there is a protective factor for women due to the small sample size and the possibility of selection bias. The Ala97Ser patients showed late onset– almost all were over 50 years of age. It differed from Val30Met patients, which showed early or late onset [24]. The course from the onset to final diagnosis ranged from 0 to 13 years, while there was no significant correlation between this and the severity of clinical manifestations. Numbness of the lower or upper extremities was among the most common initial symptoms.

**Table 2:** Clinical presentations of the probands with Ala97Ser (p.Ala117Ser) ATTRv-PN in various reports

References	Lai et al.	Yang et al.	Liu et al.	Chao et al.	Tachibana et al.	Klein et al.	Chen et al.	Yuan et al.	Our study
Origin	the Taiwan area, China	the Taiwan area, China	the Taiwan area, China	the Taiwan area, China	the Taiwan area, China	USA	Mainland China	Mainland China	Mainland China
Gender (M/F)	14/4	16/3	3/2	25/3	1/0	1/0	1/0	1/0	18/3
Age of onset (years)	65.2 ± 5.4	59.5 ± 5.7	51.2	59.9 ± 6.0	68	64	68	55	56.5 ± 7.2
Weakness	NA	19 (19)	5 (5)	28 (28)	+	NA	+	+	15 (21)
Paresthesia	NA	19 (19)	5 (5)	28 (28)	+	NA	−	+	21 (21)
Allodynia	NA	11 (19)	NA	15 (28)	−	NA	−	−	10 (21)
Autonomic dysfunction	NA	19 (19)	5 (5)	22 (28)	+	NA	+	+	18(21)
Cardiac dysfunction	NA	NA	3 (5)	NA	+	NA	+	NA	17 (21)
Gastrointestinal symptoms	NA	18 (19)	5 (5)	NA	+	NA	+	+	13 (21)
Renal dysfunction	NA	NA	1 (5)	NA	NA	NA	NA	−	4(21)
Ocular dysfunction	NA	NA	NA	NA	NA	NA	NA	−	4(21)

“+”with the symptom, “−” without the symptom, NA: not applicabl

Due to its insidious onset, there is usually a delay in the diagnosis of this disease, which makes it difficult to treat. Apart from peripheral nerve dysfunction, autonomic dysfunction was also particularly prominent. Constipation was more common than orthostatic hypotension, and it (constipation) often manifested as alternating between diarrhea and constipation. Remarkably, almost all males had erectile dysfunction at an early stage, which is an important feature that differentiates them from female patients. Cardiac dysfunction was the most common involvement of other organs. One of the 21 patients died of a cardiac incident shortly after the diagnosis. A unique phenotype of ATTRv-PN has been reported in a case report describing a distinctive chronic dry cough [16]. In our study, eight patients had similar symptoms. It occurs at different stages of the disease.

Further research is needed to confirm this mechanism. Although nerve biopsy helped diagnose the disease, not all patients in our study underwent a nerve biopsy. Only 11/15 (73.3%) patients showed positive Congo red staining. Patients with ATTRv-PN negative Congo red staining might get misdiagnosed. Interestingly, Neurophysiological examinations [25], vagus nerve ultrasonography [26], neurofilament light chain [27], and skin biopsy [28] also helped with the diagnosis. The final diagnosis was based on genetic and pathological examinations. In the absence of definitive amyloid deposits, a definitive diagnosis needs to be made in the context of the previously described clinical manifestations of multisystem involvement, genetic evidence, and the exclusion of other diseases.

In addition, many factors, such as the rarity of the disease in the general population, physicians’ lack of understanding of various clinical features, and limited diagnostic tools (i.e., histopathology and gene screening), lead to a high misdiagnosis rate and poor prognosis. ATTRv-PN is often misdiagnosed as chronic idiopathic axonal polyneuropathy, chronic inflammatory demyelinating polyneuropathy, and lumbar spinal stenosis. Diabetes or chronic alcoholism may cause polyneuropathies similar to ATTRv-PN. It may also be misdiagnosed as Charcot-Marie-Tooth or motor neuron disease. Delayed diagnosis is the main obstacle to the optimal management of ATTRv-PN in China. There is usually an interval of several years from the initial clinical symptoms of the disease to the final diagnosis. Because of the significant unmet medical needs for this rare and fatal disease, there is an urgent need to raise disease awareness to facilitate early diagnosis and timely treatment.

Historically, mutations in the *TTR* gene have predominantly been associated with symptoms affecting the peripheral nerves and cardiac system. Consequently, existing literature has visually represented the proportion of peripheral nerve and cardiac impairments across various mutation sites, facilitating people’s understanding of the clinical manifestations associated with different *TTR* gene mutation locations. However, it is important to note that *TTR* gene mutations can also impact other organs and systems, including the kidneys, eyes, and autonomic nervous system. Our study introduces a more comprehensive radar chart for multidimensional assessment, enhancing the graphical representation of clinical symptoms associated with different *TTR* gene mutation sites.

Nonetheless, our study’s sample size remained insufficient for robust statistical analysis, and all cases were sourced exclusively from southern China. Consequently, these findings may not be representative of the rest of China or the world, thereby constraining the generalizability and precision of the results.

Moreover, this study was conducted retrospectively, which introduced challenges such as incomplete data and selection bias. Notably, there was a disproportionate male-to-female ratio among the participants, and a significant delay was observed between the onset of symptoms and the final diagnosis. The retrospective design further precluded the possibility of patient follow-up, resulting in a deficiency of information regarding patient treatment and prognosis. These limitations may have impeded the precise assessment and comprehensive understanding of the disease under investigation.

Although the *TTR* gene was examined in all participants of this study, other potential genetic variants and gene-environment interactions were not analyzed or discussed. These aspects need further investigation in more comprehensive and detailed studies.

## Conclusion

Our findings elucidate the clinical spectrum of ATTRv-PN associated with the *TTR* Ala97Ser mutation in Southern China, underscoring the need for more extensive, multi-centric studies to fully delineate the disease’s impact.

## Electronic supplementary material

Below is the link to the electronic supplementary material.

Supplementary Material 1

Supplementary Material 2

## Data Availability

The data sets generated and analyzed during the current study are available from the corresponding author upon reasonable request. All data generated and analyzed during this study are included in this article.
