# Supplementary material for: Clinical features of hereditary transthyretin amyloidosis-polyneuropathy with transthyretin Ala97Ser(p.Ala117Ser) mutation in South Mainland China
Source: Orphanet J Rare Dis. 2025 Apr 28;20:202. doi: 10.1186/s13023-025-03733-0 (PMC12039301; doi:10.1186/s13023-025-03733-0)
Supplement: Supplementary file 1 — Supplementary Material 1 [file 13023_2025_3733_MOESM1_ESM.docx]

**Supplementary table 1 The clinical manifestations of the probands with Ala97Ser (p.Ala117Ser) ATTRv-PN**

| Patient No. | 1 | 2 | 3 | 4 | 5 | 6 | 7 | 8 | 9 | 10 | 11 | 12 | 13 | 14 | 15 | 16 | 17 | 18 | 19 | 20 | 21 |  |  |  |
| --- | --- | --- | --- | --- | --- | --- | --- | --- | --- | --- | --- | --- | --- | --- | --- | --- | --- | --- | --- | --- | --- | --- | --- | --- |
| *Past history* |  |  |  |  |  |  |  |  |  |  |  |  |  |  |  |  |  |  |  |  |  | *Total* |  |  |
| Hypertension | 0 | 0 | 0 | 0 | 0 | 0 | 0 | 0 | 0 | 1 | 1 | 1 | 0 | 0 | 0 | 0 | 0 | 1 | 0 | 0 | 0 | 4 |  |  |
| Diabetes mellitus | 0 | 0 | 0 | 1 | 0 | 0 | 0 | 0 | 0 | 0 | 0 | 0 | 0 | 0 | 0 | 0 | 0 | 0 | 0 | 0 | 0 | 1 |  |  |
| *Dysfunction of peripheral nerves* |  |  |  |  |  |  |  |  |  |  |  |  |  |  |  |  |  |  |  |  |  | *Total* |  |  |
| Paresthesia | 1 | 1 | 1 | 1 | 1 | 1 | 1 | 1 | 1 | 1 | 1 | 1 | 1 | 1 | 1 | 1 | 1 | 1 | 1 | 1 | 1 | 21 |  |  |
| Sensory dissociation | 1 | 0 | 0 | 1 | 0 | 0 | 0 | 0 | 1 | 0 | 1 | 0 | 0 | 0 | 0 | 0 | 0 | 0 | 1 | 0 | 0 | 5 |  |  |
| Allodynia | 0 | 0 | 1 | 1 | 1 | 1 | 0 | 0 | 0 | 0 | 1 | 1 | 0 | 1 | 0 | 1 | 0 | 0 | 1 | 1 | 0 | 10 |  |  |
| Distal weakness | 1 | 1 | 0 | 0 | 1 | 1 | 1 | 1 | 0 | 1 | 1 | 1 | 0 | 1 | 0 | 1 | 0 | 1 | 1 | 1 | 1 | 15 |  |  |
| Proximal weakness | 1 | 1 | 0 | 0 | 1 | 1 | 1 | 1 | 0 | 1 | 1 | 1 | 0 | 0 | 0 | 1 | 0 | 1 | 1 | 1 | 0 | 13 |  |  |
| Amyotrophy | 1 | 1 | 0 | 0 | 1 | 1 | 1 | 1 | 0 | 1 | 1 | 1 | 0 | 1 | 0 | 1 | 0 | 0 | 1 | 1 | 1 | 14 |  |  |
| Decreased reflexes | 1 | 1 | 1 | 1 | 1 | 1 | 0 | 1 | 1 | 1 | 1 | 1 | 0 | 1 | 1 | 1 | 0 | 0 | 1 | 1 | 1 | 17 |  |  |
| Carpal tunnel syndrome | 1 | 1 | 1 | 1 | 1 | 1 | 1 | 1 | 1 | 1 | 1 | 1 | 1 | 0 | 0 | 1 | 0 | 1 | 0 | 0 | 1 | 16 |  |  |
| *Autonomic dysfunction* |  |  |  |  |  |  |  |  |  |  |  |  |  |  |  |  |  |  |  |  |  | *Total* |  |  |
| Diarrhea | 0 | 1 | 1 | 1 | 0 | 0 | 1 | 0 | 1 | 0 | 1 | 0 | 0 | 0 | 0 | 0 | 0 | 0 | 1 | 1 | 0 | 8 |  |  |
| Constipation | 0 | 1 | 1 | 1 | 1 | 1 | 1 | 0 | 1 | 0 | 1 | 1 | 1 | 0 | 0 | 1 | 0 | 0 | 0 | 0 | 0 | 11 |  |  |
| Orthostatic hypotension | 0 | 0 | 0 | 0 | 1 | 0 | 1 | 0 | 1 | 1 | 0 | 0 | 0 | 0 | 1 | 0 | 0 | 0 | 1 | 0 | 1 | 7 |  |  |
| Hyperhidrosis | 0 | 0 | 0 | 1 | 0 | 0 | 1 | 0 | 0 | 0 | 1 | 0 | 0 | 0 | 1 | 0 | 0 | 0 | 0 | 0 | 0 | 4 |  |  |
| Erectile dysfunction | NK | 1 | 1 | NK | 1 | 1 | 1 | 0 | 1 | 1 | 1 | NA | NA | 1 | 0 | 0 | 0 | 0 | 0 | 0 | NK | 9 (18) |  |  |
| Urine retention | 1 | 0 | 0 | 0 | 0 | 0 | 0 | 0 | 0 | 0 | 0 | 0 | 0 | 0 | 0 | 0 | 0 | 0 | 1 | 0 | 0 | 2 |  |  |
| *Involvement of other organs* |  |  |  |  |  |  |  |  |  |  |  |  |  |  |  |  |  |  |  |  |  | *Total* |  |  |
| Cardiac dysfunction |  |  |  |  |  |  |  |  |  |  |  |  |  |  |  |  |  |  |  |  |  |  |  |  |
| Arrhythmia | 1 | 1 | 0 | 0 | 0 | 1 | 1 | 1 | 1 | 1 | 0 | 1 | 0 | 1 | 0 | 1 | 1 | 1 | 1 | 1 | 1 | 9 |  |  |
| Cardiac hypertrophy | 0 | 1 | 1 | 1 | 0 | 1 | 1 | 1 | 0 | 1 | 0 | 1 | 0 | 1 | 0 | 1 | 0 | 0 | 1 | 1 | NA | 12 |  |  |
| Symptomatic heart failure | 1 | 1 | 0 | 1 | 0 | 0 | 0 | 0 | 0 | 1 | 0 | 1 | 0 | 0 | 0 | 0 | 0 | 1 | 0 | 0 | 1 | 7 |  |  |
| Hepatic dysfunction | 0 | 0 | 0 | 0 | 0 | 0 | 0 | 0 | 0 | 1 | 0 | 1 | 0 | 0 | 0 | 0 | 0 | 0 | 0 | 0 | 1 | 3 |  |  |
| Renal dysfunction | 0 | 0 | 0 | 0 | 0 | 0 | 0 | 0 | 0 | 0 | 0 | 1 | 0 | 1 | 0 | 1 | 0 | 0 | 0 | 0 | 1 | 4 |  |  |
| Ocular dysfunction |  |  |  |  |  |  |  |  |  |  |  |  |  |  |  |  |  |  |  |  |  |  |  |  |
| Vision loss | 0 | 0 | 0 | 0 | 0 | 1 | 0 | 0 | NK | 1 | NK | 1 | 0 | 0 | 0 | 0 | 0 | 0 | 1 | 0 | 0 | 4 |  |  |
| Vitreous opacity | 0 | 0 | 0 | 0 | 0 | 1 | 0 | 0 | NK | NK | NK | 0 | 0 | 0 | 0 | 0 | 0 | 0 | 0 | 0 | 0 | 1 |  |  |
| Cataract | 0 | 0 | 0 | 0 | 0 | 0 | 0 | 0 | NK | NK | NK | 1 | 0 | 0 | 0 | 0 | 0 | 0 | 0 | 0 | 0 | 1 |  |  |
| Glaucoma | 0 | 0 | 0 | 0 | 0 | 0 | 0 | 0 | NK | NK | NK | 0 | 0 | 0 | 0 | 0 | 0 | 0 | 0 | 0 | 0 | 0 |  |  |
| *Others* |  |  |  |  |  |  |  |  |  |  |  |  |  |  |  |  |  |  |  |  |  | *Total* |  |  |
| Weight reduction | 0 | 1 | 1 | 1 | 1 | 1 | 1 | 1 | 1 | 1 | 1 | 1 | 0 | 1 | 0 | 1 | 1 | 1 | 1 | 0 | 1 | 17 |  |  |
| Edema | 1 | 1 | 0 | 1 | 0 | 0 | 0 | 0 | 1 | 1 | 0 | 1 | 1 | 0 | 0 | 0 | 1 | 0 | 0 | 0 | 0 | 8 |  |  |
| Dry cough | 0 | 1 | 0 | 1 | 1 | 0 | 0 | 0 | 0 | 0 | 1 | 1 | 0 | 0 | 1 | 0 | 1 | 1 | 0 | 0 | 0 | 8 |  |  |
| Hemorrhagic rash | 0 | 0 | 0 | 0 | 0 | 0 | 0 | 0 | 1 | 1 | 0 | 0 | 0 | 0 | 0 | 0 | 0 | 0 | 0 | 0 | 0 | 2 |  |  |

1: with the symptom. 0: without the symptom. NA: not applicable. NK: unknown.
