# Supplementary material for: Clinical features of hereditary transthyretin amyloidosis-polyneuropathy with transthyretin Ala97Ser(p.Ala117Ser) mutation in South Mainland China
Source: Orphanet J Rare Dis. 2025 Apr 28;20:202. doi: 10.1186/s13023-025-03733-0 (PMC12039301; doi:10.1186/s13023-025-03733-0)
Supplement: Supplementary file 2 — Supplementary Material 2 [file 13023_2025_3733_MOESM2_ESM.docx]

**Supplementary table 2 The NCS of the probands with Ala97Ser (p.Ala117Ser) ATTRv-PN**

| Patient No. | Age (years) | *DL (mS)* R/L | | *CMAP (mV)* R/L | | | | *SNAP (μV)* R/L | | | | *F wave(%)* R/L | |
| --- | --- | --- | --- | --- | --- | --- | --- | --- | --- | --- | --- | --- | --- |
|  |  | Median | Tibal | Median | Ulnar | Tibal | Peroneal | Median | Ulnar | Peroneal | Sural R/L | Median | Tibal |
| 1 | 66 | 6.1 / NR | NR / NR | 1.0 / 3.4 | 0.6 / 2.0 | NE / NE | NE / NE | NE / NE | NE / NE | NR / NR | NE / NE | 6 / 12 | 0 / 0 |
| 2 | 61 | NR / 6.3 | NE / 7.2 | NR / 1.6 | NR / 3.9 | NE / 0.5 | 0.1 / 0.4 | NR / NE | NR / NE | NE / NE | NR / NR | NR/ NR | NR / NR |
| 3 | 50 | 4.5 / NR | 4.6 / 5.2 | 4.4 / NR | 4.6 / NR | 2.5 / 1.5 | 2.0 / 1.4 | 0.3 / NR | 7.3 / NR | NR / NR | 2.2 / 2.4 | NR / NR | NR / NR |
| 4 | 66 | 4.9 / 4.8 | 5.4 / 4.6 | 5.9 / 5.1 | 5.1 / NR | 1.2 / 2.3 | 1.0 / NR | NE / NE | NE / NE | NE / NE | 0.6 / NR | ↓ / ↓ | ↓ / ↓ |
| 5 | 62 | 2.4 / 1.9 | 5.4 / 5.7 | 2.4 / 2.7 | 4.9 / 3.2 | 2.2 / 4.2 | 1.8 / 2.5 | 0.4 / NE | 0.7 / NE | NR / NR | NE / 0.4 | 100 / 0 | NR / NR |
| 6 | 62 | NE / 5.5 | NR / NR | NE / 1.1 | 2.6 / 0.9 | NR / NR | NE / NE | NE / NE | NE / NE | NE / NE | NE / NE | NR / NR | NR / NR |
| 7 | 61 | 3.9 / 3.4 | 5.7 / 3.1 | 5.4 / 7.3 | 5.1 / NR | 2.6 / 11.1 | 1.2 / NR | NE / 2.6 | NE / 2.6 | NE / NR | NR / NR | NE / NR | NR / NR |
| 8 | 67 | 3.0 / 2.8 | 8.2 / NE | 1.7 / 1.5 | 4.1 / 1.1 | 0.3 / NE | NE / NE | NE / NE | NE / 1.3 | NR / NR | NE / NE | 0 / 100 | NR / NR |
| 9 | 60 | 6.5 / 4.5 | 4.5 / 3.8 | 4.2 / 7.3 | 1.8 / 3.7 | 1.4 / 4.8 | 0.4 / 0.1 | NE / NR | NE / NR | NR / NR | NE / NR | NR / 56.3 | NR / NR |
| 10 | 65 | NR / 4.1 | NE / 4.3 | NR / 3.3 | NR / 6.9 | NE / 0.1 | 0.3 / 0.2 | NR / NE | NR / 9 | NR / NR | NE / NE | NR / 43.8 | NR / 0 |
| 11 | 59 | 6.5 / NR | NE / NE | 0.9 / NR | 2.5 / NR | NE / NE | NE / NE | NE / NE | NE / NE | NR / NR | NE / NE | 55.0 / NR | 0 / 0 |
| 12 | 68 | 5.8 / NE | 4.2 / 3.3 | 1.3 / NE | 1.6 / 3.1 | 0.5 / 2.0 | 0.1 / NE | NE / NE | NE / NE | NE / NE | NE / 1.2 | 95.0 / NR | NR / 55.0 |
| 13 | 67 | NR / 4.2 | 4.4 /5.1 | NR / 10.5 | NR / 12.7 | 3.4 / 4.2 | NE / 2.6 | NR / 10.7 | NR /11.1 | NE / NE | NE / NE | NR / NR | NR / 94.4 |
| 14 | 59 | 4.65/4.68 | 5.49/NE | 0.84/1.60 | 3.2/5.3 | 0.17/NE | 0.27/NE | NE/NE | NE/NE | NE/NE | 2.3/1.84 | NE/NE | NR/NR |
| 15 | 41 | 2.9/NR | 4.4/3.8 | 17.7/NR | 14.7/NR | 25.0/27.4 | 9.9/8.9 | 37.7/NR | 38/NR | 20.9/20.5 | 14.8/13.2 | 80/NR | 100/100 |
| 16 | 67 | 4.52/4.21 | 4.46/4.92 | 0.91/0.27 | 9.0/7.0 | 0.64/1.32 | NE/0.1 | NE/NE | NE/NE | NE/NE | 0.83/NE | NR/NR | 5/NR |
| 17 | 40 | 3.65/3.96 | 4.58/3.71 | 16.4/15.0 | 19.0/13.7 | 25.4/28.2 | 6.9/8.4 | 34.1/30.1 | 14.9/10.4 | 2.8/4.1 | 10.8/17.4 | 50/NR | 100/NR |
| 18 | 72 | NR/NR | NR/NR | NR/NR | NR/NR | NR/NR | NR/NR | NR/NR | NR/NR | NR/NR | NR/NR | NR/NR | NR/NR |
| 19 | 65 | 4.80/4.74 | NE/NE | 4.9/3.4 | 4.7/4.2 | NE/NE | 0.52/NE | NE/4.1 | NE/NE | NE/NE | NE/NE | 100/NR | NR/NR |
| 20 | 68 | 4.56/4.19 | 4.88/4.03 | 2.3/2.0 | 6.3/7.2 | 0.6/1.77 | NE/0.86 | NE/1.94 | 0.41/1.39 | NE/NE | NE/NE | 35/15 | 100/87.5 |
| 21 | 60 | 5.11/5/48 | 5.38/4.57 | 1.79/3.5 | 6.1/7.2 | 0.78/0.12 | NE/0.68 | NE/NE | 2.9/NE | NE/NE | NE/1.48 | 15/NR | 25/NR |
| Normal value | 55 - 64 | ＜4.1 | ＜5.1 | ＞7 | ＞7 | ＞4 | ＞3 | ＞12.7 | ＞6.9 | ＞0.6 | ＞1.6 | ＞73 | ＞80 |
|  | 65 - 74 | ＜4.2 | ＜5.1 | ＞6 | ＞7 | ＞4 | ＞3 | ＞10.7 | ＞6.8 | ＞0.5 | ＞1.2 | ＞73 | ＞80 |

NCS: nerve conduction studies. DL: distal latency. CMAP: compound motor action potential. SNAP: sensory nerve action potential.

NR: not recordable. NE: not elicited. ↓: decreased (the specific value was unknown).
